# Measuring the dosage of brief and skill-targeted social-emotional learning (SEL) activities in humanitarian settings

**DOI:** 10.3389/fpsyg.2022.973184

**Published:** 2023-01-25

**Authors:** Zezhen Wu, Lindsay Brown, Ha Yeon Kim, Hirokazu Yoshikawa, J. Lawrence Aber

**Affiliations:** Department of Applied Psychology, New York University, New York, NY, United States

**Keywords:** social-emotional learning, skill-targeted activity, implementation, dosage, humanitarian setting

## Abstract

**Introduction:**

In humanitarian settings, social-emotional learning (SEL) programs for children are often delivered using a field-feasible approach where the programs are more easily deployable and adaptable in the field, require minimal training, and depend less on the strict sequence and structure of the program components to elicit the intended treatment effect. However, evidence is lacking on what aspects of this implementation approach enable the SEL programming to be more beneficial to children’s SEL development.

**Method:**

In this study, we propose and evaluate measures for three dimensions of dosage (quantity, duration, and temporal pattern) of two sets of brief and skill-targeted SEL activities (*Mindfulness* and *Brain Games*) implemented in 20 primary schools in two low-income chiefdoms of Sierra Leone.

**Results:**

We find preliminary evidence of predictive validity that these dosage measures could predict children’s attendance and classroom adaptive behavior.

**Discussion:**

This study is the first to develop procedures to measure the dimensions of dosage of brief SEL activities in humanitarian settings. Our findings illuminate the need for future research on optimizing the dosage and implementation design of SEL programming using brief SEL activities.

## Introduction

Wars and diseases have shattered many children’s lives. As conflicts and crises continue, promoting children’s learning and well-being through schooling becomes even more challenging in humanitarian settings (Winthrop and Kirk, 2008; UNICEF, 2021). One way to tackle this challenge is to develop and deliver programs that foster students’ social-emotional learning (SEL) in classrooms (Greenberg et al., 2003). Not only do SEL programs improve individual students’ abilities to cope with social and emotional challenges (Durlak et al., 2010; Weissberg et al., 2015), but classrooms infused with SEL-principled practices also provide a nurturing environment for students to socialize and to “cope and hope” (Winthrop and Kirk, 2008).

Over the past 20 years, numerous studies conducted in Western, high-income countries have demonstrated that school-based SEL programs can positively impact children’s social-emotional skills and academic outcomes over time (Durlak et al., 2010, 2011; Weissberg et al., 2015). However, there is a dearth of evidence on what SEL programs could support children’s development in humanitarian settings. SEL programs developed in Western contexts are typically comprehensive, pre-packaged, and lesson-based curricula to be implemented in a formal school setting with extensive support from research- and practice-oriented organizations (Durlak et al., 2011; Jones et al., 2021). These can hardly be achieved in countries plagued with conflicts and crises. First, fragile formal education systems common in humanitarian settings are unlikely to support comprehensive SEL curricula (Global Education Monitoring Report., 2018). Second, many students are unable to attend the programs regularly due to various risk factors in their lives (Kearney et al., 2019). For instance, in a set of large-scale SEL-infused remedial programs by the International Rescue Committee (IRC), the average monthly attendance rate in the program was only 50% in Lebanon and 64% in Niger (Aber et al., 2021). Third, more comprehensive programs are often difficult to “implement with fidelity” at scale (Durlak, 1998; Carroll et al., 2007)—an umbrella term for the degree to which the program is implemented as intended by the developer (Dusenbury et al., 2003). Evidence suggests that low fidelity is often associated with a loss in program effectiveness (Dane and Schneider, 1998; Dusenbury et al., 2003), and it is especially difficult to maintain fidelity for comprehensive SEL programs in humanitarian settings due to factors such as lack of trained and well-supported personnel, attrition of personnel over time, and under-resourced facilities (Murray et al., 2014).

To address these challenges, many international organizations have attempted to build their own SEL programs using a *field-feasible* approach. This approach ensures that the programs are more easily deployable and adaptable in the field, require minimal training, and depend less on the strict sequence and structure of the program components to elicit the intended treatment effect (Jones et al., 2017). One notable effort in building such an approach is the brief and skill-targeted *SEL activities* developed by the IRC and implemented through the Education in Emergencies—Evidence for Action (3EA) initiative in multiple countries in the Middle East and Africa. Specifically, these brief activities are simple, small, and essential elements of larger, more complex evidence-based SEL practices that are designed to be flexibly implemented daily in classrooms by the teachers. These activities are being proposed as additions to IRC’s SEL-infused academic programs to provide key support for children’s growth and behavioral change (Embry and Biglan, 2008). Teachers are trained to use a menu of locally-adapted activities and can flexibly choose any activity that best suits their student’s needs in each session. Although these features allow the implementation of the program to be field-feasible, the drawback to this approach is that there are likely as many specific versions of the program as teachers due to the variations in actual quantity, duration, and repetition patterns of the implemented activities. As a result, it is unclear what aspects of this implementation approach enable the SEL programming to be more beneficial to children’s SEL development, if at all (Kim et al., in press).

Our study aims to explore and illustrate one possible way to better understand and improve the effectiveness of these brief and skill-targeted SEL activities by examining their implementation dosage in two low-income chiefdoms of Sierra Leone. Specifically, we focus on measuring three dimensions of dosage: “how much, how often, and for how long” the activities were implemented (Dolan, 2018), and examining the relationship between these fine-grained measures and children’s outcomes—attendance and classroom adaptive behavior—to provide preliminary evidence of predictive validity for these measures.

### Defining and measuring the dosage of brief and skill-targeted SEL activities

Because skill-targeted SEL activities are designed to be brief and repeatable, they have the potential benefit of being implemented frequently over time. However, as teachers choose what they deem suitable to the children’s needs and preferences, it necessarily leads to great variations in the implementation *dosage* across teachers and classrooms.

Broadly, dosage describes how much of the program is delivered (Durlak, 1998). Common measures of program dosage as delivered include the program duration, number of program components, and comprehensiveness of the content [see Durlak and DuPre (2008) for a review of implementation factors]. Recent dosage frameworks of educational programs and behavioral interventions further expand the definition of dosage to encompass “how much, how often, and for how long” each set of activities in a program is implemented (Voils et al., 2012; Dolan, 2018). For brief SEL activities, these frameworks help us distinguish the dimensions of dosage at the *activity* level (e.g., number of brief SEL activities) from the ones at the *temporal* level (e.g., minutes to deliver one SEL activity session). Building on these past frameworks, we develop detailed measures to specifically capture three dimensions of dosage: *quantity* (how much), *duration* (for how long), and *temporal pattern* (how often).

First, *quantity* is measured as (1) the number of implemented activities (*amount*) and (2) the number of unique activities (*variety*) in a given period (e.g., per day, week, or month), regardless of what SEL domains the activities are targeting. An intuitive rationale for focusing on *amount* is that more activities may indicate more SEL skills that the children could receive and have the opportunity to practice if they attend the program regularly. This *amount* measure is also the most commonly used one in other implementation frameworks to represent the dosage of behavioral interventions (Voils et al., 2012; Dolan, 2018). The rationale to focus on *variety* is that more variety may indicate more diversity in the implemented activities, and less variety may indicate more repetition. On the one hand, diverse activities may provide children with more varied opportunities to expand their skill sets, while they also risk overwhelming the children with too many skills to acquire or may be more difficult for the teachers to implement. On the other hand, a smaller set of activities may provide children with more opportunities to regularly practice the targeted skill sets but reduce opportunities for them to try new and varied activities.

Next, *duration* is measured as the length of an activity session. It should not be confused with the duration of the entire program, which is more useful for cross-program comparisons but less so when schools implement the program under a relatively similar time frame (e.g., one school year). Although the activities are designed to be brief, a very short average implementation duration may indicate less adherence to the intended duration or potentially less engagement from teachers to implement the activities comprehensively. In general, we would expect that the activities best engage children when their duration is close to the duration intended by the program developer.

Finally, *temporal pattern* refers to the longitudinal repetition patterns in implementing different activities. In this paper, we focus on the *domain-specific temporal pattern* in implementing activities from groups targeting different SEL domains. Specifically, we are interested in measuring (1) how often activities targeting the same SEL domain are repeated and (2) how many activities are implemented before at least one activity is attempted from each available SEL domain. The former reflects the tendency to implement activities targeting the same skills consecutively, and the latter reflects the tendency to implement activities targeting various skills consecutively.

How activities targeting the same or different types of skills are repeated is central yet unique to the frequently implemented brief activities and thus has been less studied in past implementation frameworks. However, the concept of meaningful repetition of classroom practices is deeply rooted in the literature on establishing norms and regularities over time (Seidman, 1988; Sarason, 1996). In educational settings, norms can be created through “intentional, deliberate, frequent actions” (Jones and Bouffard, 2012). By engaging students in everyday SEL activities, teachers create norms that shape and routinize their SEL practices and habits. In a minimally-resourced classroom in humanitarian settings, these norms become especially important to create a sense of stability and predictability for students (Rawlings Lester et al., 2017) and increase their feelings of “security and control” (Cummings, 2000; Winthrop and Kirk, 2006). Furthermore, meaningful iterations of multiple SEL activities may create a “spiral curriculum” (Harden, 1999) when previously learned SEL skills are reinforced and deepened in a patterned and structured way. Therefore, it is important to capture what routines or patterns are created in repeating activities targeting various SEL domains.

### The current study

In the current study, we operationalize the three measures of dosage—quantity, duration, and temporal pattern—for two sets of brief SEL activities conducted in an IRC program called *Learn Safe in Bo* in Sierra Leone. We also take a descriptive and exploratory approach to “identify and narrow the universe of (dosage) values” (Voils et al., 2014) by analyzing the implementation data. That is, we use the measures to predict children’s outcomes in the program to acquire evidence of predictive validity (Spear, 2014).

First, we examine whether dosage measures of brief SEL activities have enough variations across the classrooms that participated in the program. Second, we examine the relationship between the measures of dosage and children’s later classroom attendance rate, adjusting for their current attendance rate.^1^ Third and finally, we examine the relationship between the measures of dosage and changes in children’s classroom adaptive behavior (concentration problems, disruptive behavior, and prosocial behavior) from the beginning to the end of the school year.

#### Research questions

RQ1: Do measures of the dimensions of dosage—(a) quantity, (b) duration, (c) temporal pattern of brief SEL activities have enough variations across classrooms?
RQ2: Do measures of the dimensions of dosage—(a) quantity, (b) duration, (c) temporal pattern—of brief SEL activities predict children’s later attendance (next day, week, month), adjusting for concurrent attendance and baseline child characteristics?
RQ3: Do measures of the dimensions of dosage—(a) quantity, (b) duration, (c) temporal pattern—of brief and skill-targeted SEL activities predict children’s classroom adaptive behaviors at the end of the school year, adjusting for children’s behavior and characteristics at baseline?

## Materials and methods

### Context

For many decades, sub-Saharan African countries have faced tremendous challenges due to armed conflict (Moe, 2009) and public health crises. Among those countries, Sierra Leone experienced an 11-year civil war (Gberie, 1998), and later an Ebola pandemic affected the lives of tens of thousands of people (World Health Organization., 2015). Large-scale studies found a high prevalence of mental health and developmental problems among Sierra Leonean children, even many years after the armed conflict (Behrendt, 2008; Yoder et al., 2016; Thulin et al., 2020).

Despite their potential to buffer Sierra Leonean children from their social and emotional challenges, SEL programming was not introduced to the country’s education system until very recently (Boisvert, 2017). Among the efforts to introduce SEL to such humanitarian settings, the IRC, in collaboration with the Ecological Approaches to Social Emotional Learning (EASEL) Lab at Harvard University and Global TIES for Children at New York University (NYU), developed and adapted several brief and skill-targeted SEL activities in Sierra Leone and other countries such as Niger and Lebanon (Brown et al., 2019, 2022; Dolan et al., 2021). In 2017–2018, the IRC implemented an SEL-infused academic program called *Learn Safe in Bo* in 20 primary schools in two chiefdoms (Baoma and Niawa Lenge) in Bo Town, Bo district, the second-largest city in Sierra Leone with a population of over 200,000, which was severely affected by the Ebola pandemic in 2014–2015.

### Program characteristics

#### Sample

Data collection was conducted in 20 schools in Baoma and Niawa Lenge. Each school had one classroom in each grade, and the study sample included all children (*N* = 1,414, 52.5% female) from all classrooms from grades one to three (*J* = 60). 40.6% of the sampled children were in the 1st grade, 33.2% in the 2nd grade, and 26.2% in the 3rd grade. There were altogether 74 teachers on record, but data collection challenges prevented reliable tracking of their names and IDs.

#### Intervention

The program had multiple teacher training and coaching components on literacy curricula and SEL activities for classroom use, material provision and facility improvement in school, and community mobilization. Two sets of brief SEL activities were implemented as part of *Learn Safe in Bo*. The first set included 24 teacher-led *Mindfulness* activities that involved various brief breathing techniques and self-regulatory strategies to help children down-regulate and relieve their stress and overwhelming emotions (Scholastic, 2011; Kim et al., 2019). The IRC developed these activities, drawing references from existing practices of mindfulness (Greenberg and Harris, 2012) and the activities in *Mindup* (Scholastic, 2011)---a comprehensive mindfulness-based SEL program for children from pre-kindergarten to eighth grade. Two recent studies in sub-Saharan countries have also found positive effects of mindfulness-based SEL programs on reducing sadness dysregulation and aggressive responses in social conflict situations for children in grades two to four in Niger^2^ (Kim et al., 2019) and more empathic behaviors and better grades for children in grade five to seven in Uganda (Matsuba et al., 2020).

There were three types of targeted skills among the 24 Mindfulness activities: (1) *discovering* (students discover what is happening around them and in their bodies), (2) *experimenting* (students build an understanding of belly breathing and the purpose of mindfulness), and (3) *accepting* (students remain still and quiet for a longer time and learn to accept the different feelings and sensations in their bodies, as well as what is happening around them).

The second set of activities included 20 teacher-led *Brain Games* activities (Jones et al., 2019). These activities were developed based on the core activities in a larger SEL program called SECURe (Social, Emotional, and Cognitive Understanding and Regulation in education) (Jones et al., 2014). These activities aim to improve children’s executive function and self-regulation. A recent cluster-randomized study delivered 40 weeks of Brain Games (five games per week) to low-income Latinx children from pre-K through fourth grade in the U.S. Although the actual quantity of implementation was not ideal (from 72 in fourth grade to 157 games in pre-K), the study still yielded marginal positive effects on regulation-related behaviors, attention control, and impulsivity (Barnes et al., 2021).

There were also three types of targeted skills (*Brain Games Power*) among the 20 *Brain Games* activities: (1) *focus* (attention skills; e.g., “The teacher says, “I spy with my little eyes something that is —” (choose a color or shape to describe an object in the room) and children look and point at what they think the object is.”), (2) *remember* (working memory; e.g., “Students stand in a circle. One by one, each student says their name and does a motion along with it. The rest of the class then repeats the name with the motion as a group, ultimately trying to remember and repeat all names and motions.”), and (3) *stop and think* (inhibitory control; e.g., “Students follow the teacher’s directions and movements, but only when the teacher says “Simon says” first.”) (Jones et al., 2019).

After conducting each activity, teachers were instructed to conclude by asking children to briefly reflect on (1) what they noticed, (2) what they felt compared to before the activity, and (3) how and when they could use the activity in their daily life. This post-game debrief was intended to draw children’s awareness of any changes experienced by the activities and increase the probability of using them outside of a classroom setting.

As a part of their training, teachers were told to conduct at least one *Mindfulness* activity and one *Brain Games* activity per day throughout the school year (see Supplementary Appendix: SEL activity list). The suggested schedule was one *Mindfulness* activity before the first class in the morning and one *Brain Games* activity before the first class in the afternoon. Teachers were instructed to conduct these activities in English, the language in which they were originally designed. Both activity sessions were expected to take around 10 min, including the brief reflection period. For both sets of activities, teachers were also instructed to try as many activities from all groups as they deemed fit.

#### Teacher training and material adaptation

Six face-to-face teacher training workshops on *Mindfulness* (two workshops) and *Brain Games* activities (four workshops) were delivered by IRC pedagogical coaches and NYU research staff in 2016–2017 in Baoma and Niawa Lenge. The teacher training workshops explained the rationale of brief SEL activities and demonstrations from the training staff on their processes.

Before holding those workshops, the research staff paid visits to five schools (three for *Mindfulness* and two for *Brain Games*) to pilot the activities among small groups of children, each with approximately 20 children. The purpose of these contextualization sessions was to refine materials for the population and ensure the materials were more readily accessible to the children. The final adaptations to the materials include (1) clarifications in the names and meaning of activities (e.g., to put on a “Mindfulness Hat” was changed to wear a “Mindfulness Cap”; The Pickler game, where one child had to attempt to make another child laugh was renamed ‘The Comedian’ since the children did not have familiarity with clowns) and (2) refinement in the prompts (e.g., new questions were added to help with reflection: “What are the differences between before you started the activity and now?”).

### Measures

#### Dosage measure: Quantity

To better understand which activities were conducted and at what frequency, teachers recorded every day whether they conducted a *Mindfulness* or *Brain Games* activity, which one it was, and why they selected that activity. Quantity was thus measured using data from these daily activity trackers. Specifically, we created two measures of quantity using this information: (1) the total number of any SEL activities (*amount*) and (2) the number of unique SEL activities (*variety*) implemented within each time frame (week, month, or school year). We calculated these measures for any SEL activity instead of each one separately because teachers implemented one *Mindfulness* and one *Brain Games* activity in 94.5% of the days (as compared to 2.7% where there was one, and 2.8% where there was none).

#### Dosage measure: Duration

The duration of the activities was collected as a part of the teacher observation protocol, with records of the length of each SEL activity session as observed by the pedagogical coaches during their monthly mentoring visits.

#### Dosage measure: Temporal pattern

We also used data from the daily activity trackers to calculate two measures of temporal patterns across groups of activities. Individual activities fell into two pre-determined activity groups by the IRC, each with three levels: (1) type of *Mindfulness* activities (discovering, experimenting, accepting) and (2) type of *Brain Games* powers (focus/attention, remember/working memory, stop and think/inhibitory control). Hence, we calculated measures of temporal patterns for these activity groups that clustered the activities by design. All measures were calculated for the entire school year to reflect the change and temporal patterns over the full implementation period. In doing so, we considered the large gaps of no schooling for all schools during the Christmas holiday (December--January) and the presidential election (March--April).^3^

The first measure is termed “Average Exhaustive Gap” (AEG), such that it refers to the number of activity gaps before all activity groups are exhausted. AEG was derived from the *Coupon* measure (Ginsburg and Karpiuk, 1994), named after the “coupon collector’s problem,” and originally developed as a simple measure of randomness in randomly generated numbers by a human. By calculating an AEG score for a given set of activity groups, we could use it to represent the average number of activity groups implemented before all the possible alternatives were implemented. For example, in a hypothetical three-activity-group set where each group targets a different SEL skill (e.g., for Brain Games, 1 = focus, 2 = remember, 3 = stop and think), “1, 1, 2, 3, 1, 1, 1, 3, 2, 1, 2, 3” would produce a (4 + 5 + 3)/3 = 4 AEG score (Towse and Neil, 1998; see Table 1 for a visual explanation). It means that, on average, four activities are implemented before teachers conduct activities from all groups. Hence, a larger AEG score would mean that more repetitions of certain activity groups happened before all possible activity groups were implemented.

**TABLE 1:** Measures of dosage (quantity, duration, and temporal pattern).

Measures of dosage	Sub-category	Operational definition	Examples/Illustrations
Quantity	Amount	Number of implemented SEL activities	✩△□○□△○ ✩○□ Amount = 10
	Variety	Number of unique SEL activities implemented in a certain period	✩△□○□△○ ✩○□ Variety = 4 (✩△□○)
Duration	–	Average length of an activity session	Activity session duration is observed and recorded by learning coaches (e.g., 10 min).
Temporal pattern	Average exhaustive gap (AEG)	Number of activity gaps before all activity groups are exhausted	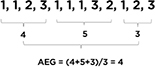 1–3: three different activity groups A larger AEG score means that more repetitions of certain activity groups happened before all possible activity groups were implemented.
	Average repetition gap (ARG)	Average activity gap between repeated activity groups	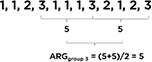 1–3: three different activity groups A larger ARG score indicates a lower tendency to repeat a given group of activities.

We created three average AEG scores (fall semester before Christmas 09/25/2017–12/03/2017; spring semester until election 01/08/2018–03/01/2018; spring semester after election 04/16/2018–06/01/2018) and then averaged these scores in each classroom. We could not calculate an AEG score among individual activities because only 6 (out of 60) classes went through all 24 *Mindfulness* activities, and 16 (out of 60) went through all 20 *Brain Games* activities.

The second measure is called Average Repetition Gap (ARG; Ginsburg and Karpiuk, 1994). This simply denotes the average gap or lag between repeated activity groups. For example, in a hypothetical three-activity-group set where each group targets a different SEL skill (e.g., for Brain Games, 1 = focus, 2 = remember, 3 = stop and think), “1, 1, 2, 3, 1, 1, 1, 3, 2, 1, 2, 3” would produce a (5 + 5)/2 = 5 ARG score for group 3 (see Table 1 for a visual explanation). In our case, it made less sense to create an overall ARG across all activities because the choice to implement one activity more frequently necessarily led to the non-implementation of others. Therefore, we created ARGs separately for the three activity groups in each classroom. For each activity group, we created three ARG scores based on the school recessions and then averaged them in each classroom. In general, higher ARG scores would indicate a lower tendency to repeat a given group of activities.

#### Attendance

Attendance is recorded from the school administrative data as reported by classroom teachers. They contained daily binary records for each child in each classroom over the school year. We also calculated the average attendance rates (sum of attended days divided by the total number of days intended) for each child per week, month, and school year.

#### Child SEL outcomes

Child SEL outcomes were collected using the Teacher Observation of Classroom Behavior-Checklist (TOCA-C) (Koth et al., 2009). TOCA-C is a teacher-report assessment of children’s socially adaptive classroom behavior. This measure contains 21 items on a six-point scale across three subscales (see Supplementary Appendix: TOCA-Checklist): Concentration Problems (seven items), Disruptive Behavior (nine items), and Prosocial Behavior (five items). The original measure was developed using a group of teachers and students from the U.S., and the reliability of the measure was good for all three subscales (Cronbach’s αs > 0.80). In our current sample, we had acceptable to good internal consistency (Concentration Problems: α_baseline_ = 0.85, α_endline_ = 0.87; Disruptive Behavior: α_baseline_ = 0.76, α_endline_ = 0.72; Prosocial Behavior: α_baseline_ = 0.68, α_endline_ = 0.66).^4^ Importantly, to minimize the reporting burden on teachers, only a randomly selected sub-sample of children (*N* = 597; around 10 children per classroom) were rated on TOCA-C by their teachers around both the baseline and the endline of the study. We calculated an average score per classroom for each of the three subscales of the TOCA-C at both time points. Of the three subscales, concentration problem and prosocial behavior are the targeted outcome of both *Mindfulness* and *Brain Games*, while disruptive behavior is not an immediate target of these activities but a medium-transfer outcome that is expected to change as children get more attentive and prosocial in classrooms.

#### Covariates

Several characteristics of the children and their households were measured using child reports in their home language at either baseline or endline (because they were assumed to be time-invariant) collected by locally-trained enumerators. These covariates could influence children’s average attendance rate and thus confound the relationship between dosage and our outcomes. The covariates include demographic characteristics (age, gender, religion, number of minutes to travel to school, number of adults in the household, number of children in the household), material well-being (material that the house was made of, number of mobile phones at home, how often hunger was felt, whether electricity was available at home), and household educational assets (parents’ job,^5^ whether the parent can read or write, how often English (the language of instruction) was spoken at home, number of books at home, how often parents talked about schoolwork, whether children helped with chores at home, and whether children helped with work outside the home).

### Analytical plans

All analyses were conducted in R (R Core Team, 2020). To answer RQ1, we examined the distribution of each measure of dosage (quantity, duration, and temporal pattern) to see if there was adequate variation across classrooms.

To answer RQ2, we examined the relationship between the measures of dosage and children’s attendance. Without a prior hypothesis on the time frame that the relationship was established, we conducted exploratory analyses using four time frames: days nested in children in classrooms, weeks nested in children in classrooms, months nested in children in classrooms, and years (no time frame) nested in children nested in classrooms. We averaged both the predictors and the outcomes at the level of each time frame and examined the coefficients on the dosage predictors (π_001_ for measures of quantity and duration; γ_001_ for measures of temporal sequence) to see if the results were sensitive to different nesting. In the analyses with time nested in classrooms, we built three-level models to account for the variation within children over time, within classrooms across children, and across classrooms (see Eqs 1–3).

In all models presented below (Eqs 1--5), *t* is the time frame (i.e., day, week, or month), and *j* is the classrooms. *X* denotes the measure of dosage for classroom j,^6^ Z denotes the *k* child-level covariates, and *Y* denotes the average attendance rate at *t*. π, β, and γ denote the random and fixed intercept coefficients at each level. ϵ, *u*, and ζ denote the error terms at each level. $\sigma_{1}^{2}$, $\tau_{1}^{2}$, and $\varphi_{1}^{2}$ denote the corresponding variances of the random effects. For models with time frames, we also included attendance lagged by one time frame as a predictor. For the models with no time frame, we built two-level hierarchical linear models with children nested in classrooms. In addition, we tested both separate models with each one of the dosage measures as a predictor and joint models with all measures as predictors. This was to understand whether there was any added value that the measures brought to explain the variance in attendance beyond each single measure, especially the most-commonly used *amount* measure in past literature. We calculated the explained variance from our linear mixed effects models using Omega-squared (Ω^2^) by Xu (2003), where $\Omega^{2}=1-\frac{\sigma^{2}}{\sigma_{0}^{2}}$ (σ^2^ is the variance of the residuals in the model, and $\sigma_{0}^{2}$ is the variance of the response variable in the data).

#### Level 1: Time


(1) $$\begin{array}{c} Y_{\left(t+1\right)\,i\,j}=\pi_{0\,i\,j}+\left(\pi_{001}\,X_{t\,i\,j}\right)+\pi_{002}\,Y_{t\,i\,j}+\epsilon_{t\,i\,j}\\ \epsilon_{t\,i\,j}∼N\,\left(0,\sigma_{1}^{2}\right) \end{array}$$

#### Level 2: Child


(2) $$\begin{array}{c} \pi_{0\,i\,j}=\beta_{00\,j}+\sum_{k}\beta_{k}\,Z_{0\,i\,j}+u_{0\,i\,j}\\ u_{0\,i\,j}∼N\,\left(0,\tau_{1}^{2}\right) \end{array}$$

#### Level 3: Classroom


(3) $$\begin{array}{c} \beta_{00\,j}=\gamma_{000}+\left(\gamma_{001}\,X_{00\,j}\right)+\zeta_{00\,j}\\ \zeta_{00\,j}∼N\,\left(0,\varphi_{1}^{2}\right) \end{array}$$

To answer RQ3, we examined the relationship between the measures of dosage and children’s classroom adaptive behavior. We used two-level hierarchical linear models with children nested in classrooms and examined the coefficients on the classroom-level dosage predictors predicting endline TOCA subscale scores (i.e., γ_01_), adjusting for baseline scores and covariates (see Eqs 4, 5). Importantly, children in the current study are nested in classrooms rather than teachers or schools. As mentioned above, this is because teachers were assigned to multiple grades and flexibly deployed on any given day, as well as staff absenteeism and turnover that were not reliably tracked. Hence, the measures of dosage were treated as classroom-level characteristics rather than being tied to each teacher’s practices. Again, we tested both separate models and joint models to examine the added value that the measures brought to explain the variance in adaptive behavior beyond each single measure.

#### Level 1: Child


(4) $$\begin{array}{c} Y_{e\,n\,d\,\_\,i\,j}=\beta_{0\,j}+\beta_{01}\,Y_{b\,a\,s\,e\,\_\,i\,j}+\sum_{k}\beta_{k}\,Z_{0\,j}+u_{0\,j}\\ u_{0\,j}∼N\,\left(0,\tau_{2}^{2}\right) \end{array}$$

#### Level 2: Classroom


(5) $$\begin{array}{c} \beta_{0\,j}=\gamma_{00}+\gamma_{01}\,X_{0\,j}+\zeta_{0\,j}\\ \zeta_{0\,j}∼N\,\left(0,\varphi_{2}^{2}\right) \end{array}$$

## Results

### RQ1: Do the measures of dosage vary across classrooms?

**FIGURE 1:**
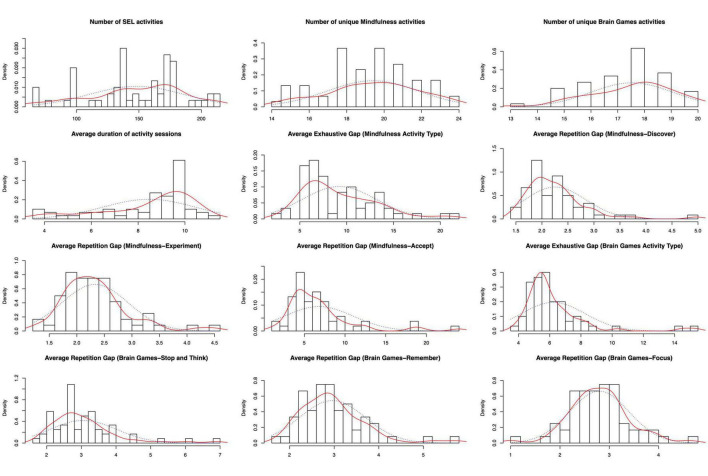
Histograms of measures of dosage across classrooms (solid lines indicating density distributions and dotted lines indicating normal fits).

Figure 1 shows the distributions of the measures of dosage across classrooms along with their density distributions (solid lines) and the normal fits (dotted lines). All measures show substantial variations across their values. AEG—the average number of activity groups implemented before all possible alternatives were attempted—was generally right-skewed. This was expected because teachers were encouraged to try a variety of activities from pre-determined groups. Therefore, classrooms are expected to produce short cycles of attempting activities from all groups. Other measures all had a wide range of values and had distributions close to normal. Pedagogical coaches reported an average activity duration of 3.67–11.20 min (*M* = 8.45, *SD* = 1.93). Because the expected duration was about 10 min, the distribution of the actual duration shows that teachers typically took less time to finish a session, and certain teachers might have implemented the activities too quickly (e.g., 4 min), possibly skipping the reflection session. The correlations are low to moderate across the measures of dosage (see Table 2).

**TABLE 2:** A correlation matrix across all measures of dosage (aggregated at the classroom level).

	1. Amount	2. Variety (Mindfulness)	3. Variety (Brain games)	4. Duration	5. AEG (Mindfulness)	6. ARG (Discovering)	7. ARG (Experimenting)	8. ARG (Accepting)	9. AEG (Brain games)	10. ARG (Stop and think)	11. ARG (Remember)	12. ARG (Focus)
1	-											
2	0.341**	-										
3	0.200	0.439***	-									
4	0.595***	0.250	0.068	-								
5	0.090	-0.219	-0.039	0.217	-							
6	-0.034	-0.058	0.038	0.210	-0.147	-						
7	0.196	0.229	0.148	-0.058	-0.044	-0.443***	-					
8	0.053	-0.313*	-0.19	0.085	0.330*	-0.002	-0.083	-				
9	0.467***	-0.082	0.149	0.191	0.162	-0.069	0.094	-0.025	-			
10	0.258*	0.070	0.083	0.253	-0.107	-0.035	0.060	0.069	0.141	-		
11	0.319*	-0.006	0.026	0.121	0.129	0.104	0.074	0.071	0.052	-0.111	-	
12	0.152	-0.215	0.077	0.123	0.034	0.098	0.098	0.138	0.246	-0.16	0.064	-

*p < 0.05; **p < 0.01; ***p < 0.001.

### RQ2a: Was the quantity of SEL activities associated with higher children’s attendance?

The average monthly attendance rate across all classrooms from September to June was 80.7%, ranging from 70.9% in March (due to the election) to 90.5% in October. However, monthly attendance rates varied greatly across classrooms and over time (see Supplementary Figure 1).

Table 3 displays how the number of SEL activities predicted children’s attendance rate aggregated at different time frames. In models with a time frame, more SEL activities consistently predicted higher children’s attendance rate at *t*+1 adjusting for the current attendance rate at *t*, such that one more SEL implementation was significantly associated with a 1.9% increase in daily attendance, 1.1% increase in weekly attendance, and 0.2% increase in monthly attendance. A similar relationship was also found when adjusting for other time-invariant covariates.

**TABLE 3:** The relationship between the number of SEL activities and children’s attendance at time t + 1 (both aggregated at different time frames).

	Day	Week	Month	School year	Day	Week	Month	School year
(Intercept)	0.627* [0.604; 0.650]	0.367* [0.347; 0.387]	0.179* [0.168; 0.190]	0.530* [0.465; 0.594]	0.556* [0.303; 0.808]	0.428* [0.263; 0.592]	0.206* [0.069; 0.344]	0.557* [0.432; 0.682]
Attendance rate at time *t*	0.192* [0.185; 0.198]	0.306* [0.293; 0.319]	0.607* [0.590; 0.623]	–	0.194* [0.187; 0.202]	0.349* [0.334; 0.364]	0.686* [0.668; 0.704]	–
Number of SEL activities	0.019* [0.011; 0.027]	0.011* [0.009; 0.013]	0.002* [0.001; 0.002]	−0.000 [−0.001; 0.000]	0.022* [0.014; 0.031]	0.010* [0.008; 0.012]	0.001* [0.001; 0.001]	−0.000 [−0.001; 0.000]
**Added covariates?**	**No**	**No**	**No**	**No**	**Yes**	**Yes**	**Yes**	**Yes**
AIC	53431.765	−9539.613	−7556.549	−2285.434	45827.670	−8165.567	−6936.349	−2273.657
BIC	53488.586	−9491.910	−7513.901	−2264.420	46097.719	−7939.485	−6734.844	−2136.293
Log likelihood	−26709.882	4775.806	3784.274	1146.717	−22884.835	4111.783	3497.174	1163.829
N_observations_	95,813	20,961	9,027	1,413	81,802	17,960	7,696	1,197
N_children_	1,394	1,398	1,401	–	1,180	1,185	1,188	–
N_classrooms_	60	60	60	60	60	60	60	60
Variance_children_	0.033	0.014	0.009	–	0.034	0.013	0.009	–
Variance_classrooms_	0.003	0.001	0.000	0.004	0.002	0.001	0.000	0.004
Variance_residual_	0.098	0.032	0.020	0.010	0.098	0.032	0.019	0.006

95% confidence intervals (CI) are also displayed (* indicates that the CI does not contain zero). Model 1–3 and 5–7 have time frames nested children and children nested in classrooms and include attendance rate (led by one time frame) as an outcome. Model 4 and 8 directly nests children in classrooms with no time frame.

Table 4 displays the relationship between the variety of SEL activities and children’s attendance aggregated at different time frames. In models with a time frame, more variety in SEL activities consistently predicted higher children’s attendance rate at *t*+1adjusting for the current attendance rate at *t*, such that one more unique SEL activity is associated with small (0.2–1.1%) but significant increase in attendance. A similar relationship was also found when adjusting for other time-invariant covariates.

**TABLE 4:** The relationship between the number of unique SEL activities and children’s attendance at time t + 1 (both aggregated at different time frames).

	Week	Month	School year	Week	Month	School year
(Intercept)	0.373* [0.354; 0.393]	0.174* [0.162; 0.187]	0.375* [0.201; 0.550]	0.433* [0.268; 0.598]	0.209* [0.071; 0.347]	0.405* [0.207; 0.604]
Attendance rate at time *t*	0.306* [0.293; 0.319]	0.620* [0.604; 0.637]	–	0.350* [0.335; 0.365]	0.697* [0.679; 0.714]	–
Number of unique SEL activities	0.011* [0.009; 0.012]	0.002* [0.001; 0.002]	0.003 [−0.002; 0.008]	0.009* [0.008; 0.011]	0.001* [0.001; 0.002]	0.003 [−0.001; 0.008]
**Added covariates?**	**No**	**No**	**No**	**Yes**	**Yes**	**Yes**
AIC	−9528.451	−7499.264	−2290.162	−8159.504	−6909.389	−2278.923
BIC	−9480.748	−7456.616	−2269.148	−7933.423	−6707.884	−2141.559
Log likelihood	4770.225	3755.632	1149.081	4108.752	3483.695	1166.462
N_observations_	20,961	9,027	1,413	17,960	7,696	1,197
N_children_	1,398	1,401	–	1,185	1,188	–
N_classrooms_	60	60	60	60	60	60
Variance_children_	0.014	0.009	–	0.013	0.009	–
Variance_classrooms_	0.001	0.000	0.004	0.001	0.000	0.003
Variance_residual_	0.032	0.021	0.010	0.032	0.019	0.006

95% confidence intervals (CI) are also displayed (* indicates that the CI does not contain zero). Model 1–2 and 4–5 have time frames nested children and children nested in classrooms and include attendance rate (led by one time frame) as an outcome. Model 3 and 6 directly nest children in classrooms with no time frame.

### RQ2b: Was the duration of SEL activities associated with higher children’s attendance?

There was little evidence that the average duration of SEL activity sessions predicted children’s attendance rate aggregated at any time frame. The signs of the relationship were inconsistent across different time frames, and no relationship was shown after adjusting for covariates (see Supplementary Table 1).

### RQ2c: Was the temporal pattern of SEL activities associated with higher children’s attendance?

In our models with measures of temporal pattern (AEG or ARG) as predictors, a smaller average repetition gap (ARG) in the *accepting* Mindfulness activity group significantly predicted higher attendance (γ = 0.004, *SE* = 0.002, 95% *CI* = [−0.008, 0.000], *p* = 0.038; that is, implementing one fewer activity targeting other skills in between two *Mindfulness* activities in the *accepting* group was associated with 0.4% increase in attendance), even after controlling for the number of unique individual *Mindfulness* activities (i.e., the variety; γ = 0.005, *SE* = 0.002, 95% *CI* = [−0.009, 0.000], *p* = 0.034). This might indicate that more frequent repetition of a variety of activities related to the *accepting* skill was related to higher attendance. None of the temporal pattern measures for any of the two activity groups yielded a statistically significant relationship with children’s attendance (see Supplementary Table 2). In addition, the explained variance was not substantially different in the model with only the *amount* measure (Ω^2^=0.288) vs. the model with all dosage measures (Ω^2^=0.280).

### RQ3a: Was the quantity of SEL activities associated with children’s classroom behavior?

There was little evidence that the amount of implemented SEL activities predicted any of the subscales of children’s endline classroom behavior, adjusting for baseline classroom behavior (see Supplementary Table 3 for the model and Supplementary Table 9 for summary statistics of the TOCA measure). Meanwhile, more variety in SEL activities predicted fewer concentration problems (γ = −0.063, *SE* = 0.027, 95% *CI* = [−0.115, −0.010], *p* = 0.022) and more prosocial behavior (γ = 0.060, *SE* = 0.023, 95% *CI* = [0.014, 0.106], *p* = 0.013), adjusting for their baseline scores. That is, more variety in SEL activities were associated with decreased teacher report of children’s concentration problems and increased report of prosocial behavior. These findings persisted when adjusting for child-level covariates (concentration problems: γ = −0.056, *SE* = 0.027, 95% *CI* = [−0.109, −0.002], *p* = 0.045; prosocial behavior: γ = 0.057, *SE* = 0.025, 95% *CI* = [0.009, 0.105], *p* = 0.025) (see Supplementary Table 4).

### RQ3b: Was the duration of SEL activities associated with children’s classroom behavior?

We found that a larger average duration of SEL activity was associated with increased endline prosocial behavior, adjusting for baseline prosocial behavior (γ = 0.101, *SE* = 0.042, 95% *CI* = [0.018, 0.184], *p* = 0.020). This finding persisted when adjusting for child-level covariates (γ = 0.092, *SE* = 0.045, 95% *CI* = [0.005, 0.180], *p* = 0.042). That is, a longer average activity duration was associated with larger positive changes in prosocial behavior (see Supplementary Table 5).

### RQ3c: Was the temporal pattern of SEL activities associated with children’s classroom behavior?

In our model with measures of temporal pattern (AEG or ARG) as predictors, a larger average exhaustive gap among the three groups of *Brain Games* activities significantly predicted positive changes in prosocial behavior (γ = 0.091, *SE* = 0.039, 95% *CI* = [0.014, 0.168], *p* = 0.025; that is, implementing one more activity targeting the same *Brain Games* skill before trying other skills was associated with 0.091 unit of increase in prosocial behavior on a six-point scale), even after controlling for the number of unique individual *Brain Games* activities (i.e., the variety; γ = 0.094, *SE* = 0.038, 95% *CI* = [0.019, 0.168], *p* = 0.018). This might indicate that the tendency to implement a variety of activities targeting the same SEL skill before trying all types of skills was related to more prosocial behavior. This finding also persisted when adjusting for child-level covariates (γ = 0.088, *SE* = 0.041, 95% *CI* = [0.009, 0.167], *p* = 0.037). Furthermore, no other measures of the temporal pattern for the other groups yielded a statistically significant relationship with children’s classroom behavior (see Supplementary Tables 6–8). In addition, the explained variance was not substantially different in the model with only the *amount* measure (prosocial behavior: Ω^2^=0.601; disruptive behavior: Ω^2^=0.591; concentration problems: Ω^2^=0.586) vs. the model with all dosage measures (prosocial behavior: Ω^2^=0.590; disruptive behavior: Ω^2^=0.614; concentration problems: Ω^2^=0.586).

## Discussion

There are many challenges in measuring the dosage of brief SEL activities and testing its relationship to program effectiveness in humanitarian settings. What we provided in this paper was a novel theory-informing analytical solution to these challenges by developing and testing both manifested (quantity and duration) and hidden (temporal pattern) measures of dosage in implementing brief SEL activities. These measures reflect a wide variety of information embedded in the implementation data commonly collected in SEL interventions (i.e., “how much, how often, and for how long” are the interventions conducted). Although we did not find consistent support for the claim that the new measures explained substantially more variance than the *amount* measure in our data, the new measures could still be conceptually useful for other research projects with similar structures of implementation data, depending on the measures’ practical relevance with the research project. In addition, our correlational findings also shed light on potential directions to improve the implementation of brief SEL activities, at least in the Sierra Leonean context. These results suggest that program developers and implementers who wish to improve children’s attendance and classroom adaptive behavior should consider increasing the amount and variety of SEL activities and the duration of each session.

### Dosage of SEL activities and children’s school attendance

In our exploratory analysis, we found some evidence in support of the relationship between measures of dosage and children’s school attendance rate at *t*+1, adjusting for the present attendance rate at *t*. To begin with, more SEL activities were associated with higher average children’s attendance. This finding provided partial support for the hypothesis that more SEL implementation could *lead to* more attendance, consistent with the pedagogical coach’s reflection on the program: “*Some pupils are actually coming to school because of those [SEL] games.*” Furthermore, a 0.2% increase in the monthly attendance rate was associated with just one more activity. Therefore, an addition of ten more activities per month (approximately two more activities per 5-day school week) would associate with a 2% increase in monthly attendance rate (0.10 increase in standard deviation), on top of an average rate of 80.7%. While exploratory, this result provides a promising strategy to increase attendance in humanitarian settings where student attendance fluctuates greatly (Brown et al., 2019). Further studies are needed to examine whether it was increased engagement in the classroom, improved SEL skills, or other factors that explained the relationship between implementing a certain amount of activities and increased attendance.

We also found that more variety in SEL activities was associated with higher attendance. This could be because classrooms with more diverse activities, instead of those repeating a smaller set of activities, motivated children to attend more or that children attended more often and more regularly, allowing the teachers to try new activities confidently. Moreover, implementing fewer activities targeting other skills in between *Mindfulness* activities in the *accepting* group was associated with an increase in attendance after controlling for the variety of individual *Mindfulness* activities. This further indicates that more frequent repetition of activities designed to target the same *accepting* skill might be especially related to higher attendance, even when individual activities varied in their specific content and format. Again, further studies are needed to examine how teachers’ and children’s motivation and behavior change due to the diversity of the implemented activities in a dynamic process.

### Dosage of SEL activities and children’s classroom adaptive behavior

Besides children’s attendance, we also found evidence supporting the relationship between several of the measures of dosage and children’s classroom adaptive behavior. First, more variety in SEL activities was associated with more prosocial behavior and fewer concentration problems. This generally matches the findings in Western countries that *Mindfulness* and *Brain Games* activities could promote children’s adaptive behavior by boosting their self-regulation and executive functions (Viglas and Perlman, 2018; Barnes et al., 2021). Thus, by implementing a variety of activities, children may have been exposed to and ultimately learned more skills related to these developmental skills. This could also be because classrooms with more diverse SEL activities created a better atmosphere for children to focus on learning and develop prosocial behavior.

Second, a longer average duration of SEL activities was associated with more prosocial behavior. This might be because teachers who spent more time on one SEL session implemented the post-game reflection or implemented it with higher quality, thereby producing more of the developer’s intended effects. Even though certain activities might take less time to implement on average (e.g., Belly breathing), very short sessions (e.g., around 3–5 min) may be less engaging for children or may omit key components of the activity. Although we could not make predictions beyond the sample space in which the range of duration was from 3 to 11 min, we did find that more time on each activity may bring out more intended effects, at least in the observed sample sessions in this program.

Third, implementing more activities targeting the same *Brain Games* skill before trying other skills was associated increase in prosocial behavior after controlling for the variety of individual *Brain Games* activities. This finding might be informative for developers of brief SEL activities to construct detailed instructions for teachers to repeat activities targeting the same skills in a more intentionally sequenced manner while allowing diversity among the content and format of individual activities. Nevertheless, we still need to be cautious about this finding, as it is not necessarily generalizable beyond the sample and context in the current study. In addition, we did not find any relationship between dosage and disruptive behavior. This might be because disruptive behavior was not an explicit immediate target behavior by either set of activities.

### Limitations and future directions

This study is one of the first in West Africa to study the dosage of brief SEL activities; thus, our study is limited in its generalizability. It also has various other limitations due to programming and methodological challenges. First, although we emphasized the exploratory nature of the analyses and reported all hypotheses that we tested, the statistically significant results should be interpreted with discretion. Specifically, we tested a total of 52 hypotheses and found 11 statistically significant results with a 5% Type I error rate, which was about 4 times the random chance.

Second, we could not separate the effect of *Mindfulness* activities from that of *Brain Games* activities. Because both activities were implemented on 94.5% of the days, we could only estimate their joint effect as brief SEL activities. However, we acknowledge that these two types of activities target different developmental domains, and the findings presented here might not be generalizable to stand-alone *Mindfulness* or *Brain Games* programs.

Third, the brief SEL activities in *Learn Safe in Bo* were only parts of this larger and more holistic climate-targeted intervention, with other components of teacher training, facility improvement, material provision, and community mobilization. Therefore, our results might not generalize to *Mindfulness* or *Brain Games* programs that are not a part of a climate-targeted intervention.

Fourth, all the activity trackers and outcome measures were reported by teachers. This posed major threats to the validity of the inferences we made using these measures. Activity trackers and attendance records were subject to errors as no other data source could verify their accuracy. The TOCA-C only measured teachers’ observation of children’s behavior, which might be biased by teachers’ knowledge and perceptions about their own SEL implementation with the children they were observing. We suggest that future studies create a data collection system to track the implemented daily activities more accurately and take a multi-informant measurement approach to measure children’s outcomes.

Fifth, all covariates used in our analysis were reported by children. The accuracy of these self-reports might be questionable. For instance, young children might not be familiar with “the material that the house was made of” or accurately remember “how often parents talked about schoolwork.” Again, we suggest that future studies take a multi-informant measurement approach to measure this personal and household-related information from both the children and their parents.

Sixth, the findings in this study were neither generalizable to other contexts nor other ranges of dosage values. We were also unable to ascertain the optimal dosage for a lack of information on the generalizability of the study and out-of-sample predictions. To do so, we would need data from other contexts and times. We also encourage future studies to validate these measures against more detailed measures of teacher decision-making in selecting activities and the quality of each activity session. We also encourage studies to explore what the *optimal* value of the dimensions of dosage (Voils et al., 2014)—quantity, duration, temporal pattern—should be to produce a “detectable effect” or the “best effect” (Carroll et al., 2007) of brief SEL activities on program-intended outcomes. As future implementation and experimental studies gather more evidence about the range of dosage values in different contexts and for children at different developmental stages, we can build evidence-based suggestions for teacher training.

Finally, we could not examine teachers’ role in implementing activities in our study. We could not reliably link teacher information to the implementation data due to data inconsistencies caused by flexible teacher deployment, teacher absenteeism, and teacher turnover. Therefore, the measures describe classroom-level implementation characteristics only, even if some might be linked to teachers’ characteristics, motivation, and classroom-management style, as suggested in past studies (Jones et al., 2014). We were also unable to investigate whether some teachers implemented the activities more consistently and patiently than others or if new teachers had trouble implementing the activities when they substituted for previous teachers. We were also unable to know if teacher bias resulted in high ratings of classroom adaptive behavior for certain groups of children with historically excluded backgrounds. In future studies, we need to collect more information on teachers to determine whether and how variation in dosage patterns relates to teachers’ characteristics and practices. Although we could not make strong inferences about teachers in this study, their perceptions of the brief SEL activities and their attitudes and strategies in implementing them matter greatly to the quality delivery of the activities, especially for those that require more teacher-child interactions (Donohue et al., 2000; Hromek and Roffey, 2009).

## Conclusion

In conclusion, this study is the first to develop procedures to measure the dimensions of dosage of brief SEL activities and explore their relationship with children’s outcomes in humanitarian settings. While exploratory, our findings provide a set of concrete and promising strategies that we can implement and further test to improve the implementation and effectiveness of such SEL programming. It also illuminates the need for future research on developing and validating measures of dosage to provide an evidence-based strategy for implementing these easily trainable, less costly, and effective brief SEL activities in these contexts. Brief and skill-targeted SEL activities hold promise as a feasible programming approach that can improve children’s learning and development, especially in crisis-affected low-resource settings, and context- and population-specific implementation dosage, such as those explored in this study, can be used to strengthen its impact.

## Data availability statement

Replication data supporting the conclusions of this article will be made available by the authors under a CC-BY 4.0 (Creative Commons Attribution 4.0 International license).

## Ethics statement

The studies involving human participants were reviewed and approved by International Rescue Committee Institutional Review Board # 0009752 and New York University Institutional Review Board # IRB-FY2016-1174. Written informed consent to participate in this study was provided by the participants or their legal guardian/next of kin.

## Author contributions

ZW conceived the main research questions, carried out secondary data analysis to answer these questions using data from a project led by LB at NYU Global TIES, and wrote the manuscript. LB, HK, HY, and JA provided the critical feedback and revisions for the manuscript. All authors read and approved the final manuscript.

## Footnotes

1 The direct and indirect outcomes of the brief SEL activities implemented in *Learn Safe in Bo* are associated negatively with several known risk factors for school absenteeism, such as anxiety and depression, negative school attitude and low academic self-concept (Gubbels et al., 2019), consistent with the mounting evidence on the “added value” of SEL programs in children’s schooling outcomes (Gjicali et al., 2020). Furthermore, preliminary qualitative evidence suggests that a higher dosage of SEL activities might even *lead to* a higher attendance rate among children in *Learn Safe in Bo*, as indicated in the interviews for pedagogical coaches [Brown, L. (in preparation). *Attendance patterns and predictors of attendance among primary school children in Sierra Leone*. New York, NY].

2 Kim et al. (2019) implemented in Niger the same *Mindfulness* activities that were implemented in *Learn Safe in Bo*.

3 Schools were closed in March for about a month due to security issues resulting from the national elections.

4 Item 13 and 14 were excluded in analysis for both baseline and endline because they greatly lowered the Cronbach’s α. Baseline data collection was in September 2017, and endline data collection was in June 2018.

5 Parents’ job is categorically coded: 1 = “Farming”, 2 = “Mining”, 3 = “Businessperson (seller or vendor in market)”, and 4 = “Formal employment (teacher, driver, carpenter, mechanic, etc.)”.

6 For measures of temporal patterns, we built one model that included all measures instead of separate models with each of the measures as the only predictor.
