# Supplementary material for: Measuring the dosage of brief and skill-targeted social-emotional learning (SEL) activities in humanitarian settings
Source: Front Psychol. 2023 Jan 25;13:973184. doi: 10.3389/fpsyg.2022.973184 (PMC9905149; doi:10.3389/fpsyg.2022.973184)
Supplement: Supplementary file 1 [file Data_Sheet_1.docx]

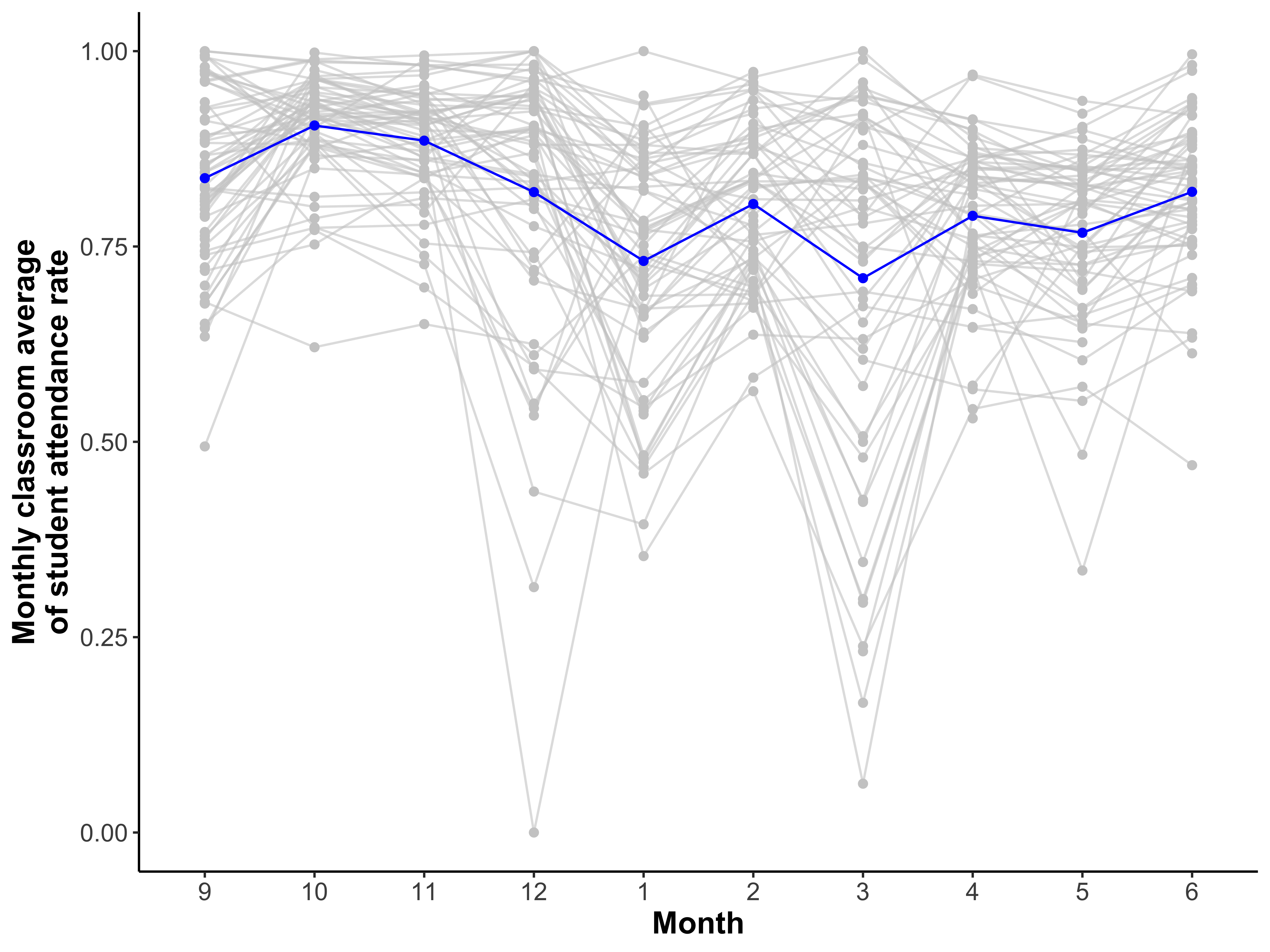


**Figure A1.**

*Monthly patterns of the class averages of student’s average monthly attendance rates*

*(Gray lines indicates trends for each of the 60 classes and the blue line indicates the overall average trend. Students’ attendance rate was calculated as the number of attended days divided by the number of days that the class was open.)*

**Table A1.**

*The relationship between the average duration of SEL activity sessions and children’s attendance at time t + 1 (both aggregated at different time frames)*

|  | **Day** | **Week** | **Month** | **School Year** | **Day** | **Week** | **Month** | **School Year** |
| --- | --- | --- | --- | --- | --- | --- | --- | --- |
| (Intercept) | 0.417^*^ | 0.523^*^ | 0.191^*^ | 0.560^*^ | 0.321^*^ | 0.584^*^ | 0.210^*^ | 0.576^*^ |
|  | [ 0.377; 0.456] | [ 0.487; 0.560] | [ 0.170; 0.212] | [ 0.489; 0.630] | [ 0.083; 0.560] | [ 0.364; 0.804] | [ 0.034; 0.387] | [ 0.446; 0.706] |
| Attendance rate | 0.463^*^ | 0.236^*^ | 0.637^*^ | -- | 0.451^*^ | 0.295^*^ | 0.716^*^ | -- |
| at time $t$ | [ 0.440; 0.485] | [ 0.207; 0.265] | [ 0.616; 0.658] | -- | [ 0.425; 0.477] | [ 0.261; 0.329] | [ 0.693; 0.738] | -- |
| Duration of SEL | 0.001 | -0.003 | 0.001 | -0.008^*^ | 0.002 | -0.002 | 0.001 | -0.006 |
| activity sessions | [-0.002; 0.005] | [-0.006; 0.000] | [-0.001; 0.003] | [-0.017; -0.000] | [-0.001; 0.006] | [-0.006; 0.001] | [-0.001; 0.003] | [-0.014; 0.003] |
| **Added covariates?** | **No** | **No** | **No** | **No** | **Yes** | **Yes** | **Yes** | **Yes** |
| AIC | 4599.593 | -709.488 | -3528.363 | -2264.136 | 3935.413 | -499.751 | -3285.970 | -2253.828 |
| BIC | 4640.704 | -671.033 | -3488.964 | -2243.193 | 4129.548 | -318.264 | -3100.078 | -2116.895 |
| Log Likelihood | -2293.797 | 360.744 | 1770.181 | 1136.068 | -1938.706 | 278.875 | 1671.985 | 1153.914 |
| N _observations_ | 6987 | 4488 | 5253 | 1388 | 5969 | 3859 | 4492 | 1178 |
| N _children_ | 1354 | 1351 | 1363 | -- | 1154 | 1150 | 1159 | -- |
| N _classrooms_ | 59 | 59 | 59 | 59 | 59 | 59 | 59 | 59 |
| Variance _children_ | 0.008 | 0.014 | 0.015 | -- | 0.009 | 0.012 | 0.013 | -- |
| Variance _classrooms_ | 0.003 | 0.003 | 0.000 | 0.003 | 0.003 | 0.003 | 0.000 | 0.004 |
| Variance _residual_ | 0.105 | 0.039 | 0.021 | 0.010 | 0.101 | 0.039 | 0.020 | 0.006 |
| *Note*. 95% confidence intervals (CI) are also displayed (* indicates that the CI does not contain zero).  Model 1-3 and 5-7 have time frames nested children and children nested in classrooms and include attendance rate (led by one time frame) as an outcome. Model 4 and 8 directly nests children in classrooms with no time frame and thus does not have lagged attendance rate as predictors. | | | | | | | | |

**Table A2.**

*The relationship between measures of temporal pattern and children’s attendance (aggregated at the classroom for the entire school year)*

| **Outcome:**  **Classroom average attendance rate** | **Mindfulness** | | | | **Brain Games** | | | |
| --- | --- | --- | --- | --- | --- | --- | --- | --- |
|  | **AEG** | **ARG**  **(Discovering)** | **ARG**  **(Experimenting)** | **ARG**  **(Accepting)** | **AEG** | **ARG**  **(Stop & think)** | **ARG**  **(Remember)** | **ARG**  **(Focus)** |
| **Panel A: Models without covariates** | | | | | | | | |
| (Intercept) | 0.517^*^ | 0.469^*^ | 0.463^*^ | 0.523^*^ | 0.501^*^ | 0.515^*^ | 0.512^*^ | 0.512^*^ |
|  | [ 0.476; 0.557] | [ 0.405; 0.534] | [ 0.397; 0.528] | [ 0.492; 0.554] | [ 0.448; 0.553] | [ 0.460; 0.570] | [ 0.445; 0.580] | [ 0.434; 0.589] |
| Slope coefficient on the predictors | -0.003 | 0.008 | 0.011 | -0.004^*^ | -0.002 | -0.009 | -0.008 | -0.009 |
|  | [-0.007; 0.001] | [-0.020; 0.036] | [-0.016; 0.038] | [-0.008; -0.000] | [-0.010; 0.006] | [-0.026; 0.008] | [-0.030; 0.014] | [-0.036; 0.019] |
| **Added covariates?** | **No** | **No** | **No** | **No** | **No** | **No** | **No** | **No** |
| AIC | -2290.547 | -2292.430 | -2292.675 | -2040.122 | -2289.878 | -2292.203 | -2292.178 | -2292.434 |
| BIC | -2269.533 | -2271.416 | -2271.661 | -2019.586 | -2268.864 | -2271.189 | -2271.164 | -2271.420 |
| Log Likelihood | 1149.274 | 1150.215 | 1150.338 | 1024.061 | 1148.939 | 1150.101 | 1150.089 | 1150.217 |
| N _children_ | 1413 | 1413 | 1413 | 1254 | 1413 | 1413 | 1413 | 1413 |
| N _classrooms_ | 60 | 60 | 60 | 53 | 60 | 60 | 60 | 60 |
| Variance _classrooms_ | 0.003 | 0.004 | 0.004 | 0.003 | 0.004 | 0.004 | 0.004 | 0.004 |
| Variance _residual_ | 0.010 | 0.010 | 0.010 | 0.010 | 0.010 | 0.010 | 0.010 | 0.010 |
| **Panel B : Models with covariates** | | | | | | | | |
| (Intercept) | 0.547^*^ | 0.508^*^ | 0.504^*^ | 0.548^*^ | 0.516^*^ | 0.535^*^ | 0.550^*^ | 0.561^*^ |
|  | [ 0.432; 0.662] | [ 0.382; 0.633] | [ 0.379; 0.628] | [ 0.428; 0.667] | [ 0.398; 0.635] | [ 0.416; 0.654] | [ 0.424; 0.676] | [ 0.428; 0.695] |
| Slope coefficient on the predictors | -0.003 | 0.005 | 0.007 | -0.004 | 0.001 | -0.005 | -0.010 | -0.014 |
|  | [-0.007; 0.001] | [-0.022; 0.033] | [-0.020; 0.034] | [-0.008; 0.000] | [-0.007; 0.009] | [-0.022; 0.012] | [-0.032; 0.012] | [-0.041; 0.012] |
| **Added covariates?** | **Yes** | **Yes** | **Yes** | **Yes** | **Yes** | **Yes** | **Yes** | **Yes** |
| AIC | -2278.740 | -2280.802 | -2280.883 | -2017.959 | -2278.215 | -2280.096 | -2281.034 | -2281.724 |
| BIC | -2141.376 | -2143.437 | -2143.518 | -1883.699 | -2140.851 | -2142.732 | -2143.669 | -2144.359 |
| Log Likelihood | 1166.370 | 1167.401 | 1167.441 | 1035.980 | 1166.108 | 1167.048 | 1167.517 | 1167.862 |
| N _children_ | 1197 | 1197 | 1197 | 1067 | 1197 | 1197 | 1197 | 1197 |
| N _classrooms_ | 60 | 60 | 60 | 53 | 60 | 60 | 60 | 60 |
| Variance _classrooms_ | 0.003 | 0.004 | 0.004 | 0.003 | 0.004 | 0.004 | 0.004 | 0.004 |
| Variance _residual_ | 0.006 | 0.006 | 0.006 | 0.006 | 0.006 | 0.006 | 0.006 | 0.006 |
| *Note.* 95% confidence intervals (CI) are also displayed (* indicates that the CI does not contain zero). Column names indicate the predictors of classroom average attendance rate. | | | | | | | | |

**Table A3.**

*The relationship between the number of SEL activities and children’s endline classroom behavior*

|  | **Concentration Problem** | **Disruptive Behavior** | **Prosocial**  **Behavior** | **Concentration Problem** | **Disruptive**  **Behavior** | **Prosocial**  **Behavior** |
| --- | --- | --- | --- | --- | --- | --- |
| (Intercept) | 2.165^*^ | 2.085^*^ | 2.089^*^ | 1.915 | 1.510^*^ | 2.378^*^ |
|  | [ 1.380; 2.949] | [ 1.523; 2.647] | [ 1.381; 2.798] | [-0.266; 4.096] | [ 0.112; 2.908] | [ 0.459; 4.297] |
| Baseline | 0.485^*^ |  |  | 0.480^*^ |  |  |
| concentration problem | [ 0.410; 0.560] |  |  | [ 0.397; 0.562] |  |  |
| Baseline |  | 0.264^*^ |  |  | 0.287^*^ |  |
| disruptive behavior |  | [ 0.187; 0.341] |  |  | [ 0.203; 0.371] |  |
| Baseline |  |  | 0.337^*^ |  |  | 0.364^*^ |
| prosocial behavior |  |  | [ 0.260; 0.414] |  |  | [ 0.277; 0.450] |
| Number of | -0.003 | -0.000 | 0.003 | -0.003 | -0.001 | 0.003 |
| SEL activities | [-0.008; 0.002] | [-0.003; 0.003] | [-0.001; 0.007] | [-0.008; 0.002] | [-0.004; 0.003] | [-0.001; 0.008] |
| **Added covariates?** | **No** | **No** | **No** | **Yes** | **Yes** | **Yes** |
| AIC | 1402.696 | 906.646 | 1234.019 | 1330.278 | 915.870 | 1197.804 |
| BIC | 1424.126 | 928.076 | 1255.449 | 1446.495 | 1032.087 | 1314.021 |
| Log Likelihood | -696.348 | -448.323 | -612.010 | -637.139 | -429.935 | -570.902 |
| N _children_ | 537 | 537 | 537 | 469 | 469 | 469 |
| N _classrooms_ | 60 | 60 | 60 | 60 | 60 | 60 |
| Variance _classrooms_ | 0.450 | 0.217 | 0.354 | 0.423 | 0.211 | 0.358 |
| Variance _residual_ | 0.608 | 0.236 | 0.440 | 0.619 | 0.237 | 0.452 |
| *Note*. 95% confidence intervals (CI) are also displayed (* indicates that the CI does not contain zero). | | | | | | |

**Table A4.**

*The relationship between the number of unique SEL activities and children’s endline classroom behavior*

|  | **Concentration Problem** | **Disruptive Behavior** | **Prosocial**  **Behavior** | **Concentration Problem** | **Disruptive**  **Behavior** | **Prosocial**  **Behavior** |
| --- | --- | --- | --- | --- | --- | --- |
| (Intercept) | 4.070^*^ | 1.152 | 0.318 | 3.429^*^ | 0.479 | 0.905 |
|  | [ 2.124; 6.017] | [-0.222; 2.525] | [-1.413; 2.050] | [ 0.680; 6.177] | [-1.351; 2.310] | [-1.553; 3.362] |
| Baseline | 0.487^*^ |  |  | 0.481^*^ |  |  |
| concentration problem | [ 0.412; 0.561] |  |  | [ 0.399; 0.563] |  |  |
| Baseline |  | 0.266^*^ |  |  | 0.290^*^ |  |
| disruptive behavior |  | [ 0.189; 0.343] |  |  | [ 0.206; 0.374] |  |
| Baseline |  |  | 0.342^*^ |  |  | 0.368^*^ |
| prosocial behavior |  |  | [ 0.265; 0.419] |  |  | [ 0.282; 0.454] |
| Number of unique | -0.063^*^ | 0.025 | 0.060^*^ | -0.056^*^ | 0.026 | 0.057^*^ |
| SEL activities | [-0.115; -0.010] | [-0.012; 0.061] | [ 0.014; 0.106] | [-0.109; -0.002] | [-0.011; 0.062] | [ 0.008; 0.105] |
| **Added covariates?** | **No** | **No** | **No** | **Yes** | **Yes** | **Yes** |
| AIC | 1393.773 | 900.072 | 1225.025 | 1322.566 | 909.448 | 1190.077 |
| BIC | 1415.203 | 921.502 | 1246.455 | 1438.783 | 1025.665 | 1306.293 |
| Log Likelihood | -691.887 | -445.036 | -607.513 | -633.283 | -426.724 | -567.038 |
| N _children_ | 537 | 537 | 537 | 469 | 469 | 469 |
| N _classrooms_ | 60 | 60 | 60 | 60 | 60 | 60 |
| Variance _classrooms_ | 0.415 | 0.209 | 0.327 | 0.402 | 0.202 | 0.340 |
| Variance _residual_ | 0.608 | 0.236 | 0.440 | 0.618 | 0.237 | 0.452 |
| *Note.* 95% confidence intervals (CI) are also displayed (* indicates that the CI does not contain zero). | | | | | | |

**Table A5.**

*The relationship between the average duration of SEL activity sessions and children’s endline classroom behavior*

|  | **Concentration Problem** | **Disruptive Behavior** | **Prosocial**  **Behavior** | **Concentration Problem** | **Disruptive**  **Behavior** | **Prosocial**  **Behavior** |
| --- | --- | --- | --- | --- | --- | --- |
| (Intercept) | 2.272^*^ | 1.778^*^ | 1.722^*^ | 2.262 | 1.331 | 2.549^*^ |
|  | [ 1.387; 3.157] | [ 1.205; 2.351] | [0.977; 2.466] | [-0.037; 4.560] | [-0.104; 2.767] | [ 0.569; 4.529] |
| Baseline | 0.486^*^ |  |  | 0.479^*^ |  |  |
| concentration problem | [ 0.410; 0.562] |  |  | [ 0.395; 0.563] |  |  |
| Baseline |  | 0.270^*^ |  |  | 0.294^*^ |  |
| disruptive behavior |  | [ 0.193; 0.347] |  |  | [ 0.210; 0.378] |  |
| Baseline |  |  | 0.334^*^ |  |  | 0.363^*^ |
| prosocial behavior |  |  | [0.255; 0.413] |  |  | [ 0.275; 0.451] |
| Duration of SEL | -0.061 | 0.035 | 0.101^*^ | -0.071 | 0.029 | 0.092^*^ |
| activity sessions | [-0.157; 0.036] | [-0.028; 0.098] | [0.018; 0.184] | [-0.167; 0.025] | [-0.035; 0.094] | [ 0.005; 0.180] |
| **Added covariates?** | **No** | **No** | **No** | **Yes** | **Yes** | **Yes** |
| AIC | 1381.074 | 878.448 | 1208.878 | 1306.874 | 888.667 | 1168.281 |
| BIC | 1402.429 | 899.803 | 1230.233 | 1422.610 | 1004.402 | 1284.016 |
| Log Likelihood | -685.537 | -434.224 | -599.439 | -625.437 | -416.333 | -556.140 |
| N _children_ | 529 | 529 | 529 | 461 | 461 | 461 |
| N _classrooms_ | 59 | 59 | 59 | 59 | 59 | 59 |
| Variance _classrooms_ | 0.455 | 0.201 | 0.334 | 0.419 | 0.196 | 0.348 |
| Variance _residual_ | 0.614 | 0.233 | 0.442 | 0.627 | 0.234 | 0.449 |
| *Note.* 95% confidence intervals (CI) are also displayed (* indicates that the CI does not contain zero). | | | | | | |

**Table A6.**

*The relationship between measures of temporal pattern and children’s endline concentration problem*

| **Outcome:**  **Classroom average attendance rate** | **Mindfulness** | | | | **Brain Games** | | | |
| --- | --- | --- | --- | --- | --- | --- | --- | --- |
|  | **AEG** | **ARG**  **(Discovering)** | **ARG**  **(Experimenting)** | **ARG**  **(Accepting)** | **AEG** | **ARG**  **(Stop & think)** | **ARG**  **(Remember)** | **ARG**  **(Focus)** |
| **Panel A: Models without covariates** | | | | | | | | |
| (Intercept) | 1.620^*^ | 2.078^*^ | 1.825^*^ | 1.482^*^ | 2.106^*^ | 1.696^*^ | 1.882^*^ | 1.026^*^ |
|  | [ 1.102; 2.137] | [ 1.302; 2.854] | [ 1.044; 2.605] | [ 1.031; 1.932] | [ 1.485; 2.728] | [ 1.018; 2.375] | [ 1.078; 2.687] | [ 0.121; 1.930] |
| Baseline  concentration problem | 0.487^*^ | 0.486^*^ | 0.487^*^ | 0.465^*^ | 0.489^*^ | 0.488^*^ | 0.487^*^ | 0.490^*^ |
|  | [ 0.412; 0.561] | [ 0.411; 0.561] | [ 0.412; 0.562] | [ 0.385; 0.545] | [ 0.414; 0.564] | [ 0.413; 0.563] | [ 0.412; 0.562] | [ 0.415; 0.565] |
| Slope coefficient on the predictors | 0.015 | -0.141 | -0.029 | 0.044 | -0.057 | 0.019 | -0.043 | 0.259 |
|  | [-0.032; 0.062] | [-0.457; 0.175] | [-0.337; 0.279] | [-0.003; 0.091] | [-0.147; 0.033] | [-0.177; 0.215] | [-0.296; 0.211] | [-0.045; 0.562] |
| **Added covariates?** | **No** | **No** | **No** | **No** | **No** | **No** | **No** | **No** |
| AIC | 1398.961 | 1394.779 | 1395.569 | 1260.552 | 1396.506 | 1396.471 | 1395.884 | 1392.858 |
| BIC | 1420.391 | 1416.209 | 1416.999 | 1281.389 | 1417.936 | 1417.901 | 1417.314 | 1414.288 |
| Log Likelihood | -694.481 | -692.389 | -692.784 | -625.276 | -693.253 | -693.236 | -692.942 | -691.429 |
| N _children_ | 537 | 537 | 537 | 477 | 537 | 537 | 537 | 537 |
| N _classrooms_ | 60 | 60 | 60 | 53 | 60 | 60 | 60 | 60 |
| Variance _classrooms_ | 0.458 | 0.454 | 0.461 | 0.462 | 0.447 | 0.461 | 0.460 | 0.437 |
| Variance _residual_ | 0.608 | 0.608 | 0.608 | 0.631 | 0.608 | 0.608 | 0.608 | 0.608 |
| **Panel B : Models with covariates** | | | | | | | | |
| (Intercept) | 1.309 | 1.878 | 1.441 | 1.825 | 1.907 | 1.425 | 1.645 | 0.643 |
|  | [-0.773; 3.392] | [-0.270; 4.026] | [-0.716; 3.599] | [-0.660; 4.309] | [-0.205; 4.018] | [-0.684; 3.534] | [-0.518; 3.807] | [-1.600; 2.886] |
| Baseline  concentration problem | 0.482^*^ | 0.481^*^ | 0.483^*^ | 0.447^*^ | 0.484^*^ | 0.483^*^ | 0.482^*^ | 0.486^*^ |
|  | [ 0.399; 0.564] | [ 0.399; 0.563] | [ 0.400; 0.565] | [ 0.359; 0.536] | [ 0.402; 0.567] | [ 0.400; 0.566] | [ 0.400; 0.565] | [ 0.403; 0.568] |
| Slope coefficient on the predictors | 0.015 | -0.179 | 0.009 | 0.044 | -0.066 | 0.013 | -0.061 | 0.268 |
|  | [-0.031; 0.062] | [-0.492; 0.134] | [-0.298; 0.316] | [-0.003; 0.090] | [-0.158; 0.025] | [-0.187; 0.213] | [-0.315; 0.193] | [-0.041; 0.576] |
| **Added covariates?** | **Yes** | **Yes** | **Yes** | **Yes** | **Yes** | **Yes** | **Yes** | **Yes** |
| AIC | 1326.541 | 1321.887 | 1323.185 | 1183.526 | 1323.587 | 1324.027 | 1323.339 | 1320.294 |
| BIC | 1442.758 | 1438.104 | 1439.402 | 1296.183 | 1439.804 | 1440.244 | 1439.556 | 1436.511 |
| Log Likelihood | -635.270 | -632.944 | -633.593 | -563.763 | -633.794 | -634.013 | -633.670 | -632.147 |
| N _children_ | 469 | 469 | 469 | 413 | 469 | 469 | 469 | 469 |
| N _classrooms_ | 60 | 60 | 60 | 53 | 60 | 60 | 60 | 60 |
| Variance _classrooms_ | 0.432 | 0.423 | 0.435 | 0.418 | 0.418 | 0.435 | 0.433 | 0.412 |
| Variance _residual_ | 0.619 | 0.619 | 0.619 | 0.633 | 0.619 | 0.619 | 0.619 | 0.619 |
| *Note.* 95% confidence intervals (CI) are also displayed (* indicates that the CI does not contain zero). Column names indicate the predictors of children’s endline concentration problem. | | | | | | | | |

**Table A7.**

*The relationship between measures of temporal pattern and children’s endline disruptive behavior*

| **Outcome:**  **Classroom average attendance rate** | **Mindfulness** | | | | **Brain Games** | | | |
| --- | --- | --- | --- | --- | --- | --- | --- | --- |
|  | **AEG** | **ARG**  **(Discovering)** | **ARG**  **(Experimenting)** | **ARG**  **(Accepting)** | **AEG** | **ARG**  **(Stop & think)** | **ARG**  **(Remember)** | **ARG**  **(Focus)** |
| **Panel A: Models without covariates** | | | | | | | | |
| (Intercept) | 2.049^*^ | 1.791^*^ | 2.149^*^ | 2.059^*^ | 2.175^*^ | 2.224^*^ | 1.952^*^ | 2.339^*^ |
|  | [ 1.666; 2.432] | [ 1.268; 2.315] | [ 1.562; 2.736] | [ 1.715; 2.404] | [ 1.728; 2.621] | [ 1.747; 2.701] | [ 1.367; 2.538] | [ 1.692; 2.986] |
| Baseline  disruptive behavior | 0.264^*^ | 0.260^*^ | 0.262^*^ | 0.210^*^ | 0.266^*^ | 0.264^*^ | 0.266^*^ | 0.263^*^ |
|  | [ 0.187; 0.341] | [ 0.182; 0.337] | [ 0.184; 0.341] | [ 0.128; 0.293] | [ 0.188; 0.343] | [ 0.187; 0.341] | [ 0.188; 0.343] | [ 0.185; 0.340] |
| Slope coefficient on the predictors | 0.003 | 0.132 | -0.029 | 0.029 | -0.017 | -0.049 | 0.040 | -0.093 |
|  | [-0.029; 0.035] | [-0.083; 0.346] | [-0.241; 0.183] | [-0.003; 0.061] | [-0.078; 0.045] | [-0.181; 0.084] | [-0.132; 0.213] | [-0.302; 0.117] |
| **Added covariates?** | **No** | **No** | **No** | **No** | **No** | **No** | **No** | **No** |
| AIC | 902.082 | 896.857 | 898.263 | 773.640 | 900.520 | 898.754 | 898.538 | 897.600 |
| BIC | 923.512 | 918.287 | 919.693 | 794.477 | 921.950 | 920.184 | 919.968 | 919.030 |
| Log Likelihood | -446.041 | -443.428 | -444.132 | -381.820 | -445.260 | -444.377 | -444.269 | -443.800 |
| N _children_ | 537 | 537 | 537 | 477 | 537 | 537 | 537 | 537 |
| N _classrooms_ | 60 | 60 | 60 | 53 | 60 | 60 | 60 | 60 |
| Variance _classrooms_ | 0.217 | 0.212 | 0.217 | 0.220 | 0.216 | 0.215 | 0.216 | 0.214 |
| Variance _residual_ | 0.236 | 0.236 | 0.236 | 0.220 | 0.236 | 0.236 | 0.236 | 0.236 |
| **Panel B : Models with covariates** | | | | | | | | |
| (Intercept) | 1.356^*^ | 1.116 | 1.447^*^ | 1.862^*^ | 1.384^*^ | 1.486^*^ | 1.362 | 1.810^*^ |
|  | [ 0.030; 2.681] | [-0.247; 2.479] | [ 0.043; 2.852] | [ 0.370; 3.353] | [ 0.037; 2.731] | [ 0.140; 2.832] | [-0.034; 2.758] | [ 0.363; 3.257] |
| Baseline  disruptive behavior | 0.287^*^ | 0.282^*^ | 0.286^*^ | 0.207^*^ | 0.287^*^ | 0.287^*^ | 0.288^*^ | 0.286^*^ |
|  | [ 0.203; 0.371] | [ 0.197; 0.366] | [ 0.200; 0.371] | [ 0.118; 0.297] | [ 0.203; 0.372] | [ 0.203; 0.371] | [ 0.203; 0.372] | [ 0.202; 0.370] |
| Slope coefficient on the predictors | 0.003 | 0.129 | -0.023 | 0.025 | 0.001 | -0.034 | 0.008 | -0.136 |
|  | [-0.029; 0.035] | [-0.087; 0.345] | [-0.236; 0.191] | [-0.008; 0.058] | [-0.063; 0.064] | [-0.170; 0.102] | [-0.166; 0.183] | [-0.348; 0.076] |
| **Added covariates?** | **Yes** | **Yes** | **Yes** | **Yes** | **Yes** | **Yes** | **Yes** | **Yes** |
| AIC | 911.534 | 906.378 | 907.736 | 781.582 | 910.205 | 908.445 | 908.170 | 906.201 |
| BIC | 1027.751 | 1022.594 | 1023.953 | 894.238 | 1026.422 | 1024.661 | 1024.387 | 1022.418 |
| Log Likelihood | -427.767 | -425.189 | -425.868 | -362.791 | -427.102 | -426.222 | -426.085 | -425.100 |
| N _children_ | 469 | 469 | 469 | 413 | 469 | 469 | 469 | 469 |
| N _classrooms_ | 60 | 60 | 60 | 53 | 60 | 60 | 60 | 60 |
| Variance _classrooms_ | 0.211 | 0.206 | 0.212 | 0.229 | 0.211 | 0.211 | 0.211 | 0.204 |
| Variance _residual_ | 0.237 | 0.237 | 0.237 | 0.213 | 0.237 | 0.237 | 0.237 | 0.237 |
| *Note.* 95% confidence intervals (CI) are also displayed (* indicates that the CI does not contain zero). Column names indicate the predictors of children’s endline disruptive behavior. | | | | | | | | |

**Table A8.**

*The relationship between measures of temporal pattern and children’s endline prosocial behavior*

| **Outcome:**  **Classroom average attendance rate** | **Mindfulness** | | | | **Brain Games** | | | |
| --- | --- | --- | --- | --- | --- | --- | --- | --- |
|  | **AEG** | **ARG**  **(Discovering)** | **ARG**  **(Experimenting)** | **ARG**  **(Accepting)** | **AEG** | **ARG**  **(Stop & think)** | **ARG**  **(Remember)** | **ARG**  **(Focus)** |
| **Panel A: Models without covariates** | | | | | | | | |
| (Intercept) | 2.563^*^ | 2.146^*^ | 2.160^*^ | 2.680^*^ | 1.967^*^ | 2.491^*^ | 2.479^*^ | 2.971^*^ |
|  | [ 2.030; 3.097] | [ 1.448; 2.844] | [ 1.420; 2.900] | [ 2.222; 3.137] | [1.369; 2.565] | [ 1.860; 3.122] | [ 1.715; 3.243] | [ 2.138; 3.805] |
| Baseline  prosocial behavior | 0.340^*^ | 0.336^*^ | 0.343^*^ | 0.335^*^ | 0.343^*^ | 0.340^*^ | 0.340^*^ | 0.342^*^ |
|  | [ 0.263; 0.417] | [ 0.259; 0.414] | [ 0.266; 0.420] | [ 0.251; 0.418] | [0.266; 0.420] | [ 0.263; 0.417] | [ 0.263; 0.418] | [ 0.265; 0.419] |
| Slope coefficient on the predictors | -0.002 | 0.184 | 0.159 | -0.008 | 0.091^*^ | 0.018 | 0.022 | -0.155 |
|  | [-0.044; 0.040] | [-0.095; 0.464] | [-0.111; 0.430] | [-0.050; 0.033] | [0.014; 0.168] | [-0.156; 0.192] | [-0.204; 0.247] | [-0.428; 0.117] |
| **Added covariates?** | **No** | **No** | **No** | **No** | **No** | **No** | **No** | **No** |
| AIC | 1231.531 | 1226.073 | 1226.472 | 1107.071 | 1225.139 | 1228.657 | 1228.143 | 1226.541 |
| BIC | 1252.961 | 1247.503 | 1247.902 | 1127.909 | 1246.569 | 1250.087 | 1249.573 | 1247.971 |
| Log Likelihood | -610.766 | -608.036 | -608.236 | -548.536 | -607.569 | -609.329 | -609.071 | -608.271 |
| N _children_ | 537 | 537 | 537 | 477 | 537 | 537 | 537 | 537 |
| N _classrooms_ | 60 | 60 | 60 | 53 | 60 | 60 | 60 | 60 |
| Variance _classrooms_ | 0.368 | 0.357 | 0.358 | 0.349 | 0.333 | 0.368 | 0.368 | 0.359 |
| Variance _residual_ | 0.440 | 0.440 | 0.441 | 0.455 | 0.440 | 0.440 | 0.440 | 0.441 |
| **Panel B : Models with covariates** | | | | | | | | |
| (Intercept) | 2.913^*^ | 2.465^*^ | 2.443^*^ | 3.838^*^ | 2.329^*^ | 2.961^*^ | 2.757^*^ | 3.292^*^ |
|  | [ 1.061; 4.766] | [ 0.573; 4.358] | [ 0.520; 4.366] | [ 1.648; 6.029] | [ 0.459; 4.199] | [ 1.098; 4.824] | [ 0.829; 4.685] | [ 1.297; 5.287] |
| Baseline  prosocial behavior | 0.368^*^ | 0.363^*^ | 0.372^*^ | 0.383^*^ | 0.369^*^ | 0.369^*^ | 0.369^*^ | 0.369^*^ |
|  | [ 0.281; 0.454] | [ 0.276; 0.450] | [ 0.286; 0.459] | [ 0.289; 0.477] | [ 0.283; 0.455] | [ 0.282; 0.455] | [ 0.282; 0.455] | [ 0.282; 0.455] |
| Slope coefficient on the predictors | -0.001 | 0.204 | 0.181 | -0.013 | 0.086^*^ | -0.022 | 0.048 | -0.130 |
|  | [-0.044; 0.042] | [-0.082; 0.491] | [-0.096; 0.459] | [-0.054; 0.029] | [ 0.004; 0.168] | [-0.204; 0.161] | [-0.185; 0.281] | [-0.416; 0.156] |
| **Added covariates?** | **Yes** | **Yes** | **Yes** | **Yes** | **Yes** | **Yes** | **Yes** | **Yes** |
| AIC | 1195.481 | 1189.725 | 1190.099 | 1068.850 | 1190.060 | 1192.529 | 1191.931 | 1190.889 |
| BIC | 1311.698 | 1305.942 | 1306.315 | 1181.507 | 1306.277 | 1308.746 | 1308.148 | 1307.106 |
| Log Likelihood | -569.741 | -566.863 | -567.049 | -506.425 | -567.030 | -568.265 | -567.966 | -567.445 |
| N _children_ | 469 | 469 | 469 | 413 | 469 | 469 | 469 | 469 |
| N _classrooms_ | 60 | 60 | 60 | 53 | 60 | 60 | 60 | 60 |
| Variance _classrooms_ | 0.375 | 0.361 | 0.362 | 0.342 | 0.345 | 0.374 | 0.373 | 0.369 |
| Variance _residual_ | 0.452 | 0.452 | 0.453 | 0.466 | 0.452 | 0.453 | 0.453 | 0.452 |
| *Note.* 95% confidence intervals (CI) are also displayed (* indicates that the CI does not contain zero). Column names indicate the predictors of children’s endline prosocial behavior. | | | | | | | | |

**Table A9.**

*Means, standard deviations, and correlations of the TOCA measure*

| Variable | ***M*** | ***SD*** | 1 | 2 | 3 | 4 | 5 |
| --- | --- | --- | --- | --- | --- | --- | --- |
|  |  |  |  |  |  |  |  |
| 1. Concentration problem (T1) | **3.047** | **1.149** | -- |  |  |  |  |
| 2. Disruptive behavior (T1) | **2.810** | **0.809** | 0.028 | -- |  |  |  |
| 3. Prosocial behavior (T1) | **4.112** | **1.009** | -0.508*** | 0.047 | -- |  |  |
| 4. Concentration problem (T2) | **3.216** | **1.147** | 0.448*** | 0.070 | -0.323*** | -- |  |
| 5. Disruptive behavior (T2) | **2.818** | **0.719** | 0.141*** | 0.390*** | -0.077 | 0.162*** | -- |
| 6. Prosocial behavior (T2) | **3.960** | **0.985** | -0.246*** | -0.067 | 0.444*** | -0.535*** | -0.036 |

*Note.* *M* and *SD* are used to represent mean and standard deviation, respectively. * indicates *p* < .05. ** indicates *p* < .01. *** indicates *p* < .001.

**Appendix: SEL activity list**

| **#** | **Mindfulness** | **Difficulty** | **Brain Games** | **Difficulty** | **Power** |
| --- | --- | --- | --- | --- | --- |
| 1 | Belly Breathing | 1 | Abracadabra | 1 | Focus |
| 2 | Body Scan | 1 | Freeze | 1 | Stop and Think |
| 3 | Lions Breath | 1 | Hot Potato! | 1 | Remember |
| 4 | Mindfulness of Feeling | 1 | I Find | 1 | Focus |
| 5 | Mindfulness of Thought | 1 | Name Game | 1 | Remember |
| 6 | Show and Tell | 1 | Silly Stories | 1 | Stop and Think |
| 7 | Super Hearing | 1 | Simon Says | 1 | Stop and Think |
| 8 | Super Smell | 1 | Telephone | 1 | Focus |
| 9 | Super Touch | 1 | What is Missing? | 1 | Remember |
| 10 | Big Person Steps | 2 | Catch that Sound | 2 | Focus |
| 11 | Cat and Cow | 2 | Categories | 2 | Stop and Think |
| 12 | Flapping Wings | 2 | Four Corners | 2 | Focus |
| 13 | Flying Bird | 2 | On My Plate | 2 | Remember |
| 14 | Growing and  Shrinking Circles | 2 | Sing it Silly | 2 | Stop and Think |
| 15 | Growing Seeds | 2 | Tailor Shop | 2 | Remember |
| 16 | Head, Shoulders, Neck | 2 | Ten Questions | 2 | Remember |
| 17 | Shake Everything Off | 2 | The Comedian | 2 | Stop and Think |
| 18 | Tighten and Relax | 2 | Sing with a Partner | 3 | Stop and Think |
| 19 | Back-to-Back Breathing | 3 | Who Has the Last Word? | 3 | Remember |
| 20 | Changing Shapes | 3 | Who is the Leader? | 3 | Focus |
| 21 | Counting Breaths | 3 |  |  |  |
| 22 | Focus on the Light | 3 |  |  |  |
| 23 | Loving Kindness | 3 |  |  |  |
| 24 | Tree | 3 |  |  |  |

**Appendix: TOCA-Checklist**

*In the last three weeks, would you say the following statements were never, rarely, sometimes, often, very often, or almost always true of this child?(Reversed coded items denoted as R).*

| **Concentration Problem** | |
| --- | --- |
| TOCA1R | Concentrates |
| TOCA3R | Pays attention |
| TOCA7R | Works hard |
| TOCA11R | Stay on task |
| TOCA13 | Is easily distracted |
| TOCA19R | Completes assignments |
| TOCA21R | Learns up to ability |
| **Disruptive Behavior** | |
| TOCA4 | Breaks rules |
| TOCA6 | Doesn’t get along with others |
| TOCA8 | Harms others |
| TOCA10 | Gets angry when provoked by other children |
| TOCA12 | Yells at others |
| TOCA15 | Fights |
| TOCA16 | Lies |
| TOCA18 | Harms property |
| TOCA20 | Teases classmates |
| **Prosocial Behavior** | |
| TOCA2 | Is friendly |
| TOCA5 | Is liked by classmates |
| TOCA9 | Shows empathy & compassion for other's feelings |
| TOCA14R | Is rejected by classmates |
| TOCA17 | Has many friends |
